# Cilomilast Ameliorates Renal Tubulointerstitial Fibrosis by Inhibiting the TGF-β1-Smad2/3 Signaling Pathway

**DOI:** 10.3389/fmed.2020.626140

**Published:** 2021-01-21

**Authors:** Man Xu, Shumin Li, Jiajia Wang, Songming Huang, Aihua Zhang, Yue Zhang, Wei Gu, Xiaowen Yu, Zhanjun Jia

**Affiliations:** ^1^Department of Endocrinology, Children's Hospital of Nanjing Medical University, Nanjing, China; ^2^Nanjing Key Laboratory of Pediatrics, Children's Hospital of Nanjing Medical University, Nanjing, China; ^3^Jiangsu Key Laboratory of Pediatrics, Nanjing Medical University, Nanjing, China; ^4^Department of Nephrology, Children's Hospital of Nanjing Medical University, Nanjing, China

**Keywords:** chronic kidney disease, cilomilast, TGF-β1, Smad2/3, renal tubulointerstitial fibrosis

## Abstract

**Background:** Renal tubulointerstitial fibrosis is the key pathological feature in chronic kidney diseases (CKDs) with no satisfactory therapies in clinic. Cilomilast is a second-generation, selective phosphodiesterase-4 inhibitor, but its role in renal tubulointerstitial fibrosis in CKD remains unclear.

**Material and Methods:** Cilomilast was applied to the mice with unilateral ureteric obstruction (UUO) and renal fibroblast cells (NRK-49F) stimulated by TGF-β1. Renal tubulointerstitial fibrosis and inflammation after UUO or TGF-β1 stimulation were examined by histology, Western blotting, real-time PCR and immunohistochemistry. KIM-1 and NGAL were detected to evaluate tubular injury in UUO mice.

**Results:** *In vivo*, immunohistochemistry and western blot data demonstrated that cilomilast treatment inhibited extracellular matrix deposition, profibrotic gene expression, and the inflammatory response. Furthermore, cilomilast prevented tubular injury in UUO mice, as manifested by reduced expression of KIM-1 and NGAL in the kidney. *In vitro*, cilomilast attenuated the activation of fibroblast cells stimulated by TGF-β1, as shown by the reduced expression of fibronectin, α-SMA, collagen I, and collagen III. Cilomilast also inhibited the activation of TGF-β1-Smad2/3 signaling in TGF-β1-treated fibroblast cells.

**Conclusion:** The findings of this study suggest that cilomilast is protective against renal tubulointerstitial fibrosis in CKD, possibly through the inhibition of TGF-β1-Smad2/3 signaling, indicating the translational potential of this drug in treating CKD.

## Introduction

Chronic kidney disease (CKD) has become a major public health problem in many countries. Almost all forms of chronic renal diseases can ultimately result in end-stage renal diseases (ESRDs), leading to a significant impact on quality of life as well as a substantial social burden (1). Renal tubulointerstitial fibrosis is characterized by the deposition of extracellular matrix (ECM), excessive accumulation of activated myofibroblasts, and infiltration of inflammatory cells (2, 3). Unfortunately, no satisfactory therapeutic strategies for inhibiting or reversing renal tubulointerstitia fibrosis are clinically available. Therefore, there is an urgent need to find new effective therapeutic drugs for renal tubulointerstitial fibrosis.

Phosphodiesterase 4 (PDE4) isozymes belong to the PDE superfamily and selectively hydrolyse 3′,5′-cyclic adenosine monophosphate (cAMP) with high affinity (4). In recent years, PDE4 inhibition has been applied to study its probable therapeutic value in the nervous system (5), respiratory system (6) and immune system (7). Furthermore, PDE4 inhibition has been found to attenuate lung fibrosis (8) and dermal fibrosis (9). Previous studies have shown that PDE4 is widely expressed in renal tubules (4). The inhibition of PDE4 has suppressive effect on tubular damage in acute renal failure (10–12). Recently, a report showed that one PDE4 inhibitor, rolipram, played an antifibrotic role in CKD possibly via acting on C/EBP-β and PGC-1α in tubular epithelial cells (13). Cilomilast is another PDE4 inhibitor that is currently being investigated in a phase III clinical trial for the treatment of chronic obstructive pulmonary disease (COPD). It has beneficial effects on COPD (14), tumors (15), acute lung injury (16), and acute kidney injury (12). Furthermore, cilomilast has been reported to attenuate pulmonary fibrosis (17). However, the effect of cilomilast on renal tubulointerstitial fibrosis has not been studied.

Transforming growth factor-β1 (TGF-β1) is an essential fibrogenic factor that plays a crucial role in the renal fibrotic process (18). Emerging evidence suggests that TGF-β1 initiates renal tubular epithelial cell transdifferentiation to myofibroblasts, enhancing collagen and fibronectin (FN) synthesis and extracellular matrix deposition (19–21). TGF-β1 receptor activation stimulates the translocation of decapentaplegic homolog 3 (Smad3) to the nucleus, where it regulates the transcription of target genes (22). Smad7 has an inhibitory effect on TGF-β1, Smad2, and Smad3 (23). The activation of TGFβ1-Smad2/3 signaling or the loss of inhibitory Smad7 triggers fibrotic cascades (24). In this study, we investigated the therapeutic effects of cilomilast on renal tubulointerstitial fibrosis using a mouse model of obstructive uropathy. Additionally, we further explored the antifibrotic action of cilomilast and its effect on regulating the TGFβ1-Smad2/3 pathway in renal fibroblasts. We found that cilomilast attenuated the development of renal tubulointerstitial fibrosis possibly by inhibiting the TGFβ1-Smad2/3 signaling pathway.

## Methods

### Animal Models of Chronic Kidney Fibrosis

**Figure 1:**
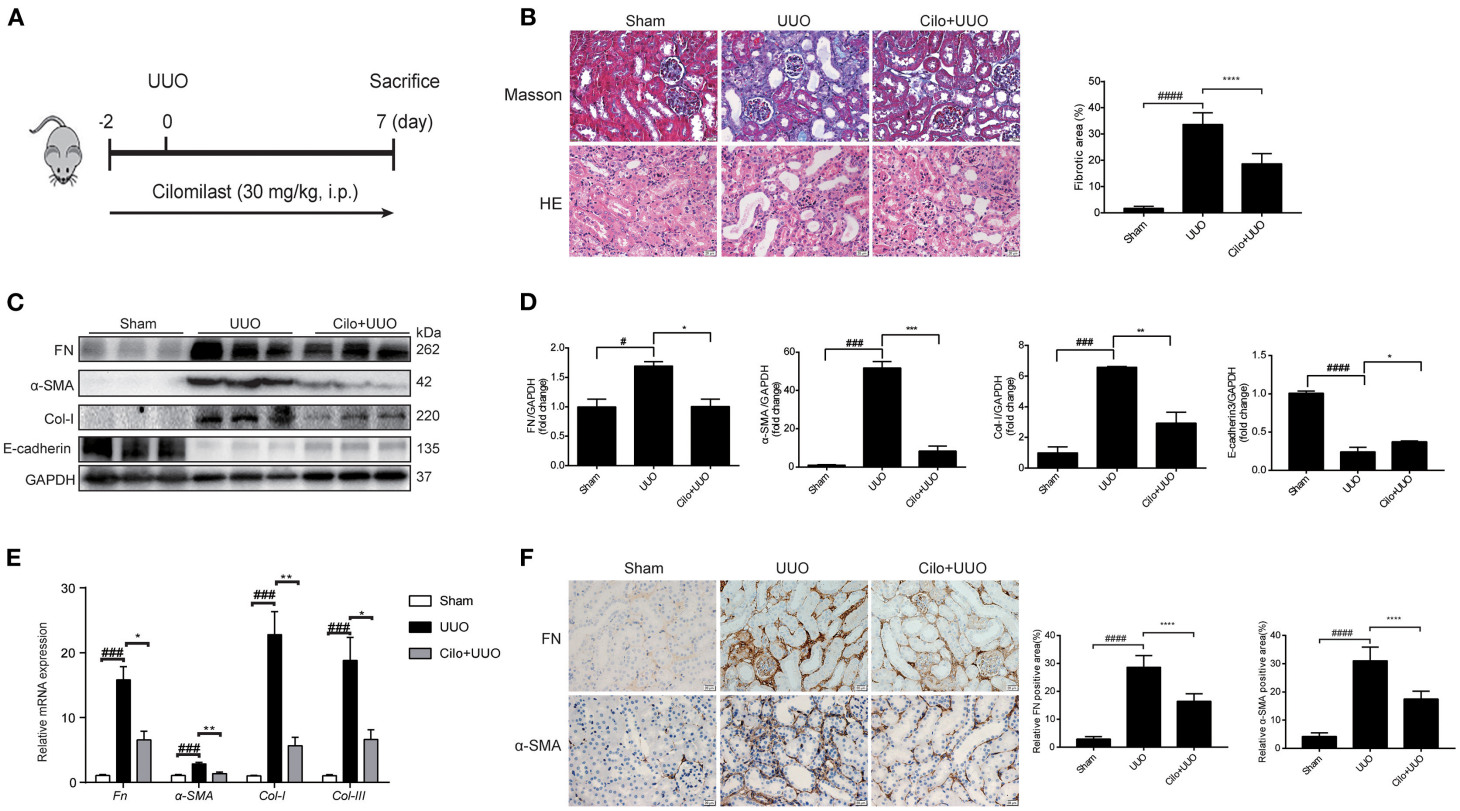
Cilomilast improved UUO-induced renal tubulointerstitial fibrosis. Mice were pre-treated with cilomilast by i.p. injection for 2 days and then treated with cilomilast by i.p. injection for 7 days after UUO surgery. Mice in the UUO group were treated with an equal volume of saline, and mice in the sham group were subjected to the same procedure without the ureteral ligation. **(A)** A schematic diagram showing the procedure of UUO-induced renal tubulointerstitial fibrosis in mice. **(B)** Masson's trichrome staining and HE staining of kidney tissue sections. Scale bar =20 μm (left). Fibrotic area in mice were analyzed (right). Six random fields were taken from each kidney. **(C)** Western blot analyses of renal FN, Col-I, α-SMA and E-cadherin protein expressions in kidney tissues. **(D)** Densitometry of the western blot results in **(C)**. **(E)** qRT-PCR analyses of *Fn, Col-I, Col-III* and α*-SMA* mRNA expression (*n* = 6). **(F)** Representative images and quantitative assessment of the expression and distribution of FN and α-SMA in kidney tissues using immunohistochemical staining. Scale bar =20 μm (left). Relative FN and α-SMA positive area in mice were analyzed (right). Six random fields were taken from each kidney. The data are presented as the mean ± SEM. Statistically significant differences were determined by one-way ANOVA and two-way ANOVA. ^#^*P* < 0.05, ^###^*P* < 0.001, ^####^*P* < 0.001, *P < 0.05, **P < 0.01, ***P < 0.001, ****P < 0.0001.

In the UUO experiment, 8-week-old male C57BL/6 mice weighing 20–25 g were divided into 3 groups (control: *n* = 6; UUO-treated: *n* = 6; and UUO+cilomilast-treated: *n* = 6). Mice were anesthetized with 2% isoflurane, and the left ureter was ligated at the ureteropelvic junction with a 4-0 silk suture through a median ventral incision. Cilomilast was intraperitoneally (i.p.) delivered to mice at 30 mg·kg^−1^·day^−1^ based on previous report (25, 26). Cilomilast or vehicle was administered to mice from −2 to 7 days before and after UUO surgery (Figure 1A). After 7 days of UUO, all mice were sacrificed by cervical dislocation and the kidney tissues were harvested for further analysis. All animal procedures were approved by the Nanjing Medical University Institutional Animal Care and Use Committee (registration number: IACUC-1809017).

### Histological Analysis

Kidney tissues were fixed in 4% PFA, embedded in paraffin, and cut into sections (4-μm-thick), which were stained with Masson's trichrome. Masson's trichrome staining was used to assess collagen deposition in the obstructed kidney tissue. Next, 8–10 randomly selected fields were observed under the microscope, and then, each mouse kidney tissue was evaluated in a blinded manner.

### Immunohistochemistry (IHC) of Animal Kidney Samples

IHC was performed as previously described (27). Briefly, paraffin-embedded animal kidney sections (4 μm) were blocked with 5% BSA for 1 h and incubated at 4°C overnight with rabbit monoclonal primary antibodies against FN (Abcam, ab2413, Cambridge, MA, USA, 1:250), α-smooth muscle actin (SMA; ab7817, Abcam, Cambridge, MA, USA, 1:400), TNF-α (ab215188, Abcam, Cambridge, MA, USA, 1:100), TGF-β1 (ab215715, Abcam, Cambridge, MA, USA, 1:500), neutrophil gelatinase-associated lipocalin (NGAL; ab63929, Abcam, Cambridge, MA, USA, 1:1000) and F4/80 (ab100790, Abcam, Cambridge, MA, USA, 1:100). After washing with TBST buffer three times, sections were incubated with horseradish peroxidase-conjugated anti-rabbit secondary antibody for 60 min. The localization of peroxidase conjugates was determined using a DAB kit (ZLI-9018, Zsbio, China). Slides were examined under a microscope, and the signals were analyzed using Image-Pro Plus software analysis tools.

### Immunofluorescence Staining

The cells were fixed with PBS containing 4% PFA for 30 min. After blocking with 5% BSA for 1 h, the slides were incubated overnight at 4°C with an anti-FN antibody (#26836, CST, Danvers, MA, USA, 1:250), which was diluted with 5% BSA overnight at 4°C. Subsequently, the cells were incubated with anti-rabbit secondary antibodies (ab150077, Abcam, Cambridge, MA, USA, 1:250) for 1 h at room temperature and were then stained with the nuclear-specific stain DAPI (Beyotime Institute of Biotechnology) for 3 min at room temperature. Then, the cells were washed three times in PBS and imaged. The slides were viewed with a Carl Zeiss LSM 5 PASCAL laser scanning confocal microscope.

### Cell Culture and Treatments

NRK-49F cells were obtained from the American Type Culture Collection (ATCC, Manassas, VA). DMEM and fetal bovine serum were purchased from Wisent Corporation (Wisent, Canada). The cells were grown in DMEM supplemented with 10% fetal bovine serum (GIBCO), penicillin (100 U/mL) and streptomycin (100 μg/mL) and maintained at 37°C in 5% CO_2_ in a humidified incubator. The cells were grown to 80% confluence. Cells were pre-treated with cilomilast (5 μM) for 1 h, and TGF-β1 (5 ng/mL) was added to 2% fetal bovine serum medium to stimulate NRK-49F cells for 24 h. In a separate exprement, NRK-49F cells were pre-treated with cilomilast (5 μM) and SB-431542 (1 μM) (a inhibitor of Smad2/3) for 1 h, and then TGF-β1 (5 ng/mL) was added to stimulate cells for 24 h.

### RNA Isolation and Quantitative Real-Time PCR (qRT-PCR)

Total RNA was extracted from kidney cortexes and cells by TRIzol (Invitrogen, Carlsbad, CA) based on the manufacturer's protocol (28). We reverse transcribed total RNA (1 μg) into cDNA using a PrimeScript™ Reverse Transcriptase System. Quantitative real-time PCR was subsequently carried out with SYBR Green Master Mix (Vazyme) on a QuantStudio 3 Real-time PCR System (Applied Biosystems, Foster City, CA). The cycling programme consisted of a preliminary denaturation (95°C for 10 min) followed by 40 cycles of 95°C for 15 s and 60°C for 1 min. Relative mRNA levels were normalized to the levels of GAPDH and calculated with the comparative threshold cycle (ΔΔCt) method. The primer sequences are shown in Table 1.

**Table 1:** Primer sequences for qRT-PCR.

**Gene (species)**	**Primer sequence**
FN (Mice)	F: GGACCTCCTCATCTACATTCG
	R: GTTCCCTCCACAGTTCAAAAG
α-SMA (Mice)	F: CCACCGATCCAGACAGAGTAC
	R: TCCACGAAACCACCTATAACA
Col-I (Mice)	F: CTCAAGGTCACGGTCACGAAC
	R: CCTGGCAAAGACGGACTCAAC
Col-III (Mice)	F: GGACCAGGCAATGATGGAAAAC
	R: GGACCAGGGAAACCCATGACA
TGF-β1 (Mice)	F: CTGAGTGGCTGTCTTTTGA
	R: TGGAGTTTGTTATCTTTGCTG
KIM-1 (Mice)	F: TCAGCTCGGGAATGCACAACC
	R: CTCCAGGGAAGCCGCAGAAAA
NGAL (Mice)	F: ACACTCACCACCCATTCA
	R: CACCACGGACTACAACCA
IL-1β (Mice)	F: TCGTGAATGAGCAGACAG
	R: AGAGGCAAGGAGGAAAAC
IL-6 (Mice)	F: GTCACCAGCATCAGTCCCAAG
	R: CCCACCAAGAACGATAGTCAA
TNF-α (Mice)	F: CAGACCCTCACACTCACAAACCAC
	R: CCTTGTCCCTTGAAGAGAACCTG
Mcp-1 (Mice)	F: GTGCTGACCCCAAGAAGGAATG
	R: TGAGGTGGTTGTGGAAAAGGTAGTG
IL-18 (Mice)	F: CATGTCAGAAGACTCTTGCGTCA
	R: TTATATTCCGTATTACTGCGGTTGT
Icam-1 (Mice)	F: GTGATGCTCAGGTATCCATCCA
	R: CACAGTTCTCAAAGCACAGCG
GAPDH (Mice)	F: AAGAAGGTGGTGAAGCAGG
	R: GAAGGTGGAAGAGTGGGAGT
FN (Rat)	F: GGACCTCCTCATCTACATTCG
	R: GTTCCCTCCACAGTTCAAAAG
α-SMA (Rat)	F: GTCTCAAACATAATCTGGGTCA
	R: GATAGAACACGGCATCATCAC
Col-I (Rat)	F: GAAGCAAAGTTTCCTCCAAGA
	R: GCCCAGAAGAATATGTATCACC
Col-III (Rat)	F: GGTTTGGAGAATCTATGAATGGTGG
	R: GCTGGAAAGAAGTCTGAGGAAGG
IL-1β (Rat)	F: AGGAGAGACAAGCAACGACA
	R: CTTTTCCATCTTCTTCTTTGGGTAT
IL-6 (Rat)	F: AGTTGCCTTCTTGGGACTGATGT
	R: GGTCTGTTGTGGGTGGTATCCTC
Mcp-1 (Rat)	F: CTGTGCTGACCCCAATAAGGAA
	R: GAGGTGGTTGTGGAAAAGAGAGTG
GAPDH (Rat)	F: GGCTCTCTGCTCCTCCC
	R: CCGTTCACACCGACCTT

### Western Blotting

We lysed cells or homogenized tissues using protein lysis buffer [50 mmol/L Tris, 150 mmol/L NaCl, 10 mmol/L EDTA, 1% Triton X-100, 200 mmol/L sodium fluoride, supplemented with 1 × protease inhibitor cocktail (Roche, 04693132001)]. Then, the samples were centrifuged (14,000 rpm) at 4°C for 15 min. The supernatant was collected and the protein concentration was determined using a BCA protein assay kit (Beyotime, China). Total protein was separated by SDS-PAGE gel and transferred onto PVDF membranes. Then the membranes were blocked by TBS-T (0.1% Tween 20 in TBS) containing 5% non-fat milk for 1 h at room temperature and incubated with primary antibodies against FN (#26836, CST, Danvers, MA, USA, 1:1000), α-SMA (ab7817, Abcam, Cambridge, MA, USA, 1:1000), Collagen-I (Col-I; ab34710, Abcam, Cambridge, MA, USA, 1:1000), kidney injury molecule-1 (KIM-1; ab190696, Abcam; Cambridge, MA, USA, 1:5000), NGAL (ab63929, Abcam, Cambridge, MA, USA, 1:1000), TGF-β1 (#3711, CST, Danvers, MA, USA, 1:1000), Smad2 (#5339, CST, Danvers, MA, USA, 1:1000), Smad2/3 (#8685, CST, Danvers, MA, USA, 1:1000), p-Smad2 (#18338, CST, Danvers, MA, USA, 1:1000), Smad3 (#9513, CST, Danvers, MA, USA, 1:1000), p-Smad3 (#9520, CST, Danvers, MA, USA, 1:1000), p-Smad2/3 (#8828, CST, Danvers, MA, USA, 1:1000), Smad7 (25840-1-AP, Proteintech, Chicago, IL, USA, 1:1000), E-cadherin (#3195, CST, Danvers, MA, USA, 1:1000)and GAPDH (#3683, CST, Danvers, MA, USA, 1:1000), followed by the addition of HRP-labeled secondary antibodies (#7074, CST, Danvers, MA, USA, 1:2500). Densitometry was analyzed with ImageJ software (NIH, Bethesda, MD, USA).

### ELISA for TGF-β1

Mouse TGF-β1 in kidney tissue homogenates was evaluated by an ELISA kit (E-EL-M0044c, Elascience, China) according to the manufacturer's protocol.

### Cell Counting Kit-8 (CCK-8) Assay

Cell viability was determined by CCK-8 assay kit (KGA317, KeyGen Biotech, China) (27). Briefly, NRK-49F cells were treated with cilomilast (5–40 μM) for 24 h, and then 10 μL CCK-8 reagent was added to medium and incubated for 2 h. The absorbance was detected at 450 nm.

### Statistical Analysis

All data are presented as the means ± standard errors of the mean (SEMs). Differences between 2 groups were analyzed using two-tailed Student's *t*-test and incorporated into GraphPad Prism 6 software (GraphPad Software). ANOVA was used for comparisons among multiple groups. *P* < 0.05 was considered significant.

## Results

### Cilomilast Treatment Attenuates UUO-Induced Renal Tubulointerstitial Fibrosis

First, we used a UUO model to explore the effect of cilomilast treatment on renal tubulointerstitial fibrosis (Figure 1A). Firstly, Masson's trichrome staining showed that cilomilast treatment led to a remarkable reduction in collagen deposition. In addition, HE staining showed that cilomilast significantly reduced tubule brush border disruption and tubular atrophy, indicating the attenuation of UUO-induced tubular injury (Figure 1B). Then, western blot and qRT-PCR analysis confirmed the deceased expression of FN, α-SMA (a marker of myofibroblasts) and Col-I and a significant increase in protein levels of E-cadherin after cilomilast treatment (Figures 1C–E). Furthermore, by using immunohistochemistry, we further found cilomilast treatment markedly inhibited the UUO-induced upregulation of FN and α-SMA expression (Figure 1F). These results demonstrated that cilomilast improved renal tubulointerstitial fibrosis.

### Cilomilast Treatment Reduced TGF-β1 in the Kidneys of UUO Mice

**Figure 2:**
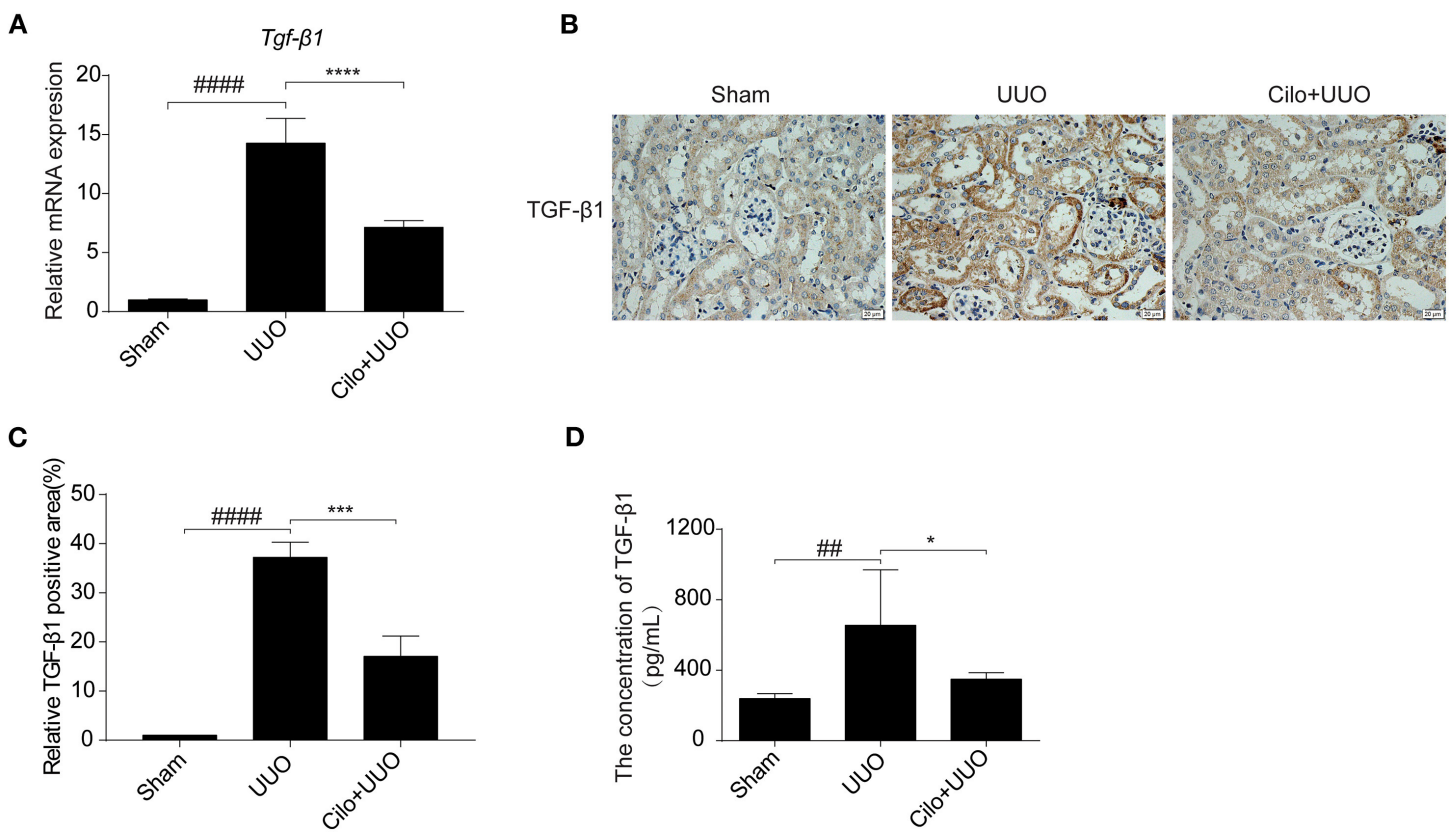
Cilomilast treatment downregulated TGF-β1 expression induced by UUO. **(A)** qRT-PCR analyses of *TGF-*β*1* mRNA expression (*n* = 6). **(B,C)** Representative images and quantitative assessment of the expression and distribution of TGF-β1 in kidney tissues using immunohistochemical staining. Scale bar = 20 μm. **(D)** ELISA analysis of TGF-β1 expression in kidney tissues. The data are presented as the mean ± SEM. Statistically significant differences were determined by one-way ANOVA. ^##^*P* < 0.01, ^####^*P* < 0.0001, *P < 0.05, ***P < 0.001, ****P < 0.0001.

TGF-β1 is involved in renal tubulointerstitial fibrosis and is produced in large quantities during renal fibrogenesis. Therefore, we examined whether the decreased fibrosis in obstructed kidneys in cilomilast-treated mice was associated with change of TGF-β1 production. By qRT-PCR assays, we found that the enhanced expression of TGF-β1 in UUO mice was significantly blunted after cilomilast treatment (Figure 2A). Furthermore, immunohistochemistry staining and ELISA also showed that the expression of TGF-β1 was significantly reduced in cilomilast-treated UUO mice (Figures 2B–D).

### Cilomilast Treatment Suppressed Renal Tubular Injury Induced by UUO

**Figure 3:**
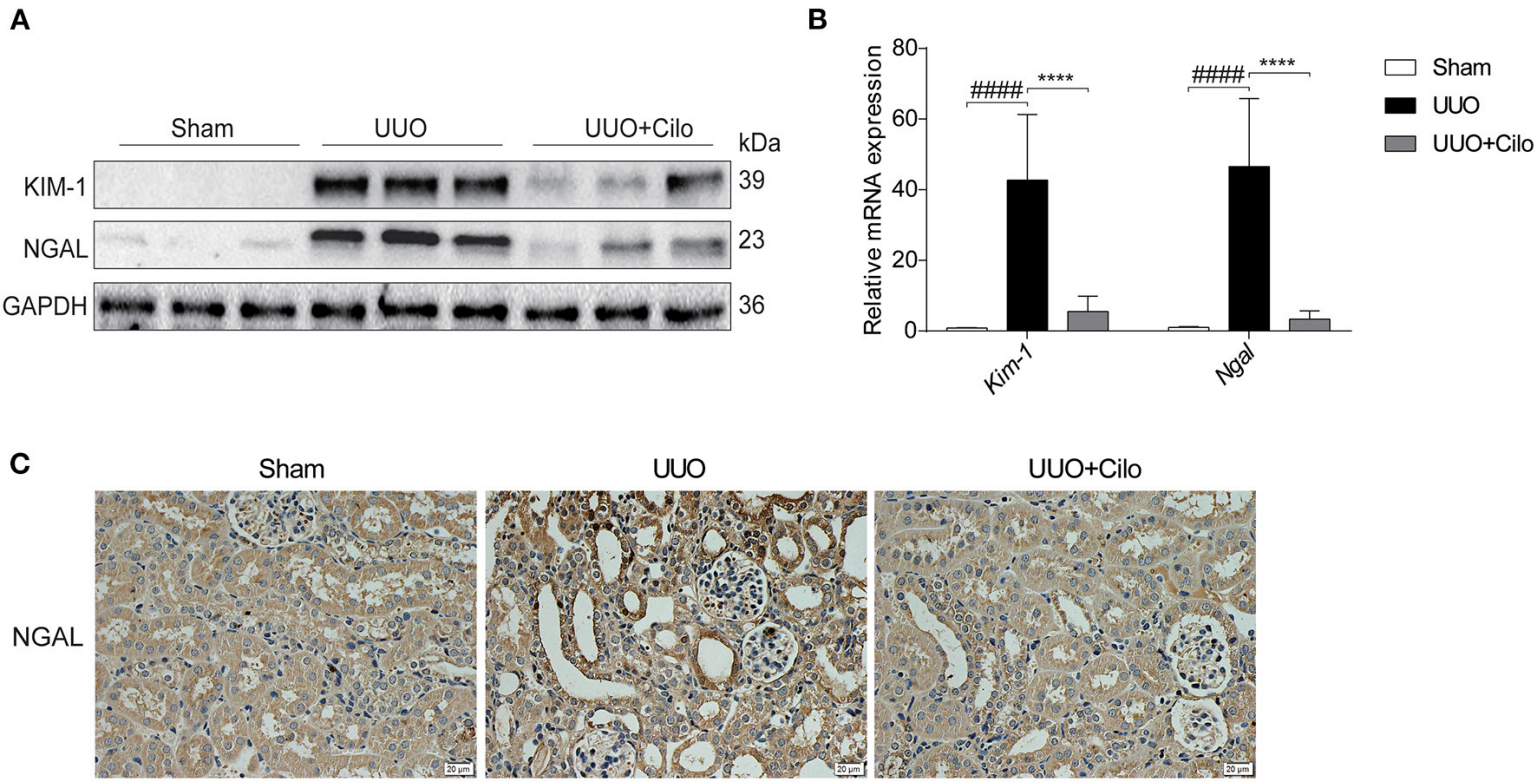
Cilomilast treatment improved renal tubular injury induced by UUO. **(A)** Western blot analyses of renal KIM-1 and NGAL protein expression in kidney tissues. **(B)** qRT-PCR analyses of *Kim-1* and *Ngal* mRNA expression (*n* = 6). **(C)** Representative images and quantitative assessment of the expression and distribution of NGAL in kidney tissues using immunohistochemical staining. Scale bar =20 μm. The data are presented as the mean ± SEM. Statistically significant differences were determined by two-way ANOVA. ^####^*P* < 0.0001, ****P < 0.0001.

To evaluate the extent of renal damage, the expression of KIM-1 and NGAL, both markers of tubular damage, was measured in the kidney. As shown in Figure 3, KIM-1 and NGAL expression was markedly increased in the UUO group according to western blot and qRT-PCR analyses, which was attenuated by cilomilast treatment (Figures 3A,B). The trend of NGAL change measured by immunohistochemistry was consistent with that of the protein and mRNA levels (Figure 3C). These data suggested that the amelioration of tubular injury after cilomilast treatment could protect tubular integrity and attenuate subsequent pathological events.

### Cilomilast Treatment Attenuated Renal Inflammation Induced by UUO

**Figure 4:**
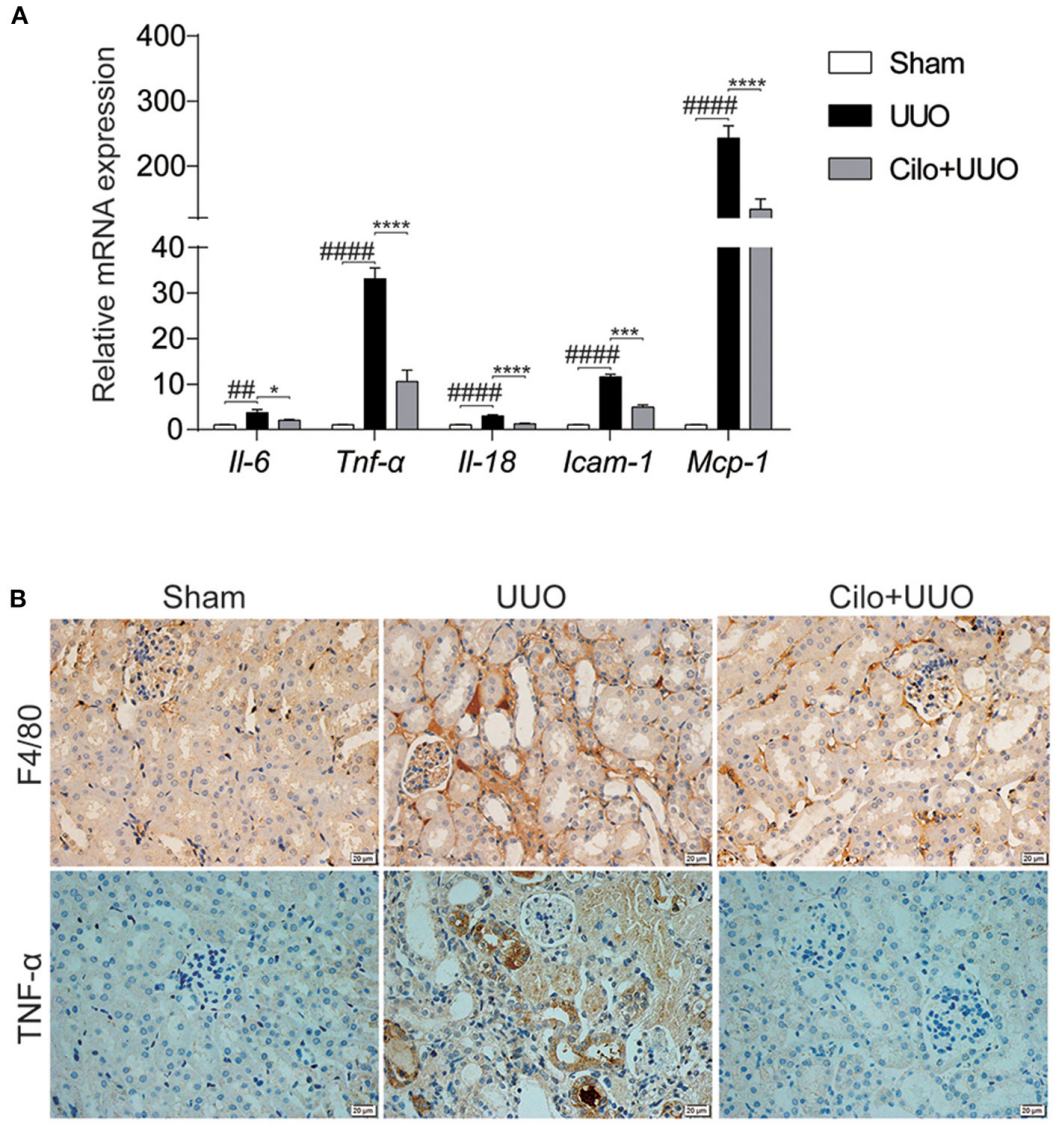
Cilomilast improved UUO-induced inflammatory responses. **(A)** qRT-PCR analyses of *Il-6, Tnf-*α, *Il-18, Icam-1*, and *Mcp-1* mRNA expression (*n* = 6). **(B)** Representative images and quantitative assessment of the expression and distribution of F4/80 and TNF-α in kidney tissues using immunohistochemical staining. Scale bar = 20 μm. The data are presented as the mean ± SEM. Statistically significant differences were determined by two-way ANOVA. ^##^*P* < 0.01, ^####^*P* < 0.0001, *P < 0.05, ***P < 0.001, ****P < 0.0001.

Inflammation plays a key role in the progression of renal tubulointerstitial fibrosis (29). Thus, we evaluated the inflammatory status (inflammatory cell infiltration and pro-inflammatory cytokine expression) in obstructed kidneys with or without cilomilast. As expected, the enhanced mRNA expression of *Il-6, Il-18, Tnf-*α, *Icam-1*, and *Mcp-1* was significantly blunted after cilomilast treatment (Figure 4A). Immunohistochemistry analysis also showed reductions in macrophage infiltration (F4/80^+^) and TNF-α expression in the obstructed kidney after cilomilast therapy (Figure 4B). These results revealed that cilomilast could ameliorate renal inflammation in UUO.

### Cilomilast Treatment Reduced the TGF-β1-Induced Profibrotic Response and Inflammation in NRK-49F Cells

**Figure 5:**
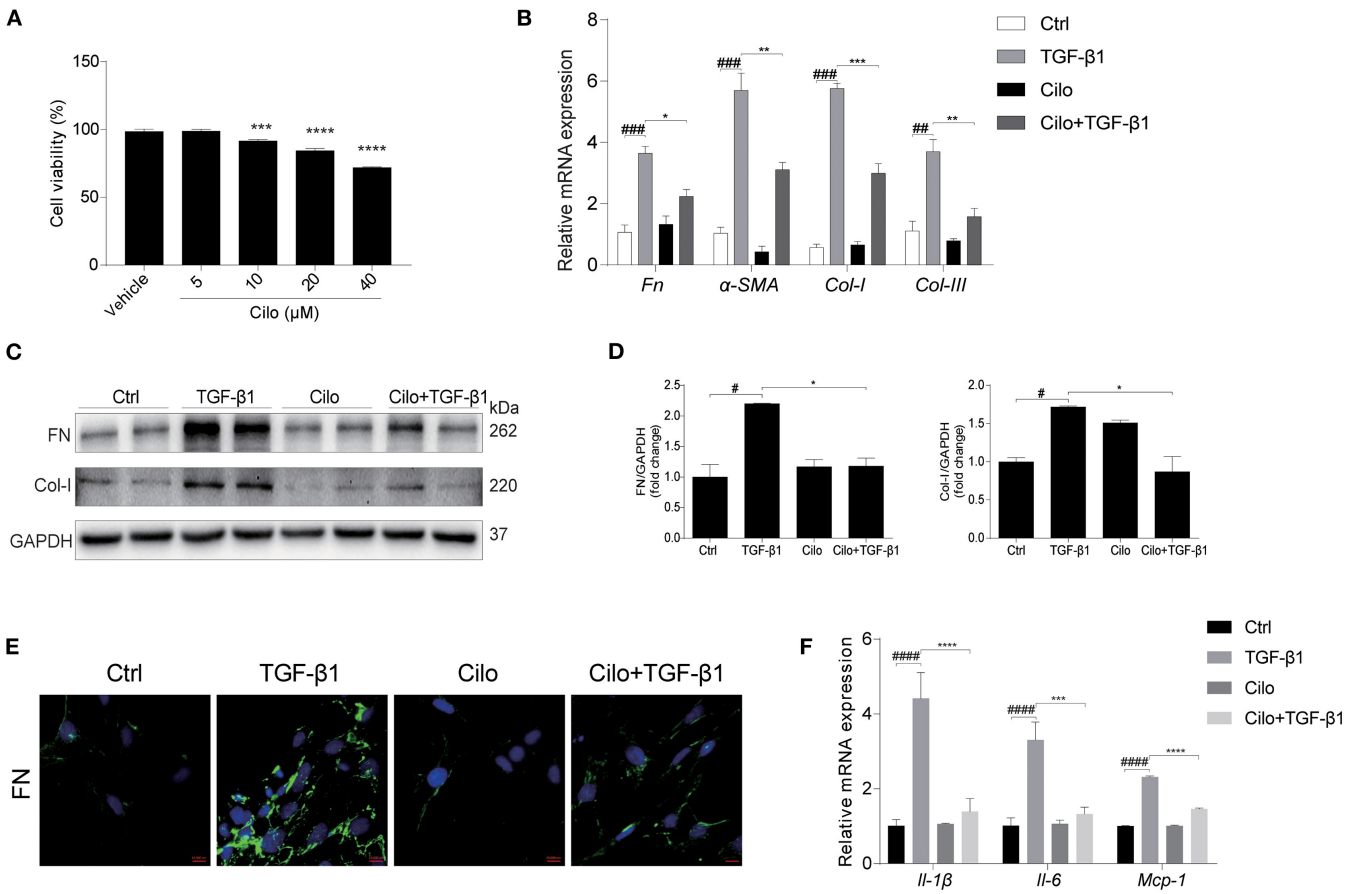
Cilomilast reduced the TGF-β1-induced profibrotic response in NRK-49F cells. NRK-49F cells stimulated with TGF-β1 were treated with cilomilast. **(A)** Cell viability was analyzed by CCK8 assay after treatment with celastrol for 24 h at increasing concentrations from 5 μM to 40 μM. **(B)** qRT-PCR analyses of *Fn, Col-I, Col-III*, and α*-SMA* mRNA expression (*n* = 3). **(C)** Western blot analyses of FN and Col-I protein expression in NRK-49F cells. **(D)** Densitometry of the western blot results in **(B)**. **(E)** Representative images of immunofluorescence staining for FN in NRK-49F cells. **(F)** qRT-PCR analyses of *Il-1*β*, Il-6*, and *Mcp-1* mRNA expression (*n* = 3). The data are presented as the mean ± SEM. Statistically significant differences were determined by one-way ANOVA and two-way ANOVA. ^#^*P* < 0.05, ^##^*P* < 0.01, ^###^*P* < 0.001, ^####^*P* < 0.0001, *P < 0.05, **P < 0.01, ***P < 0.001, ****P < 0.0001.

To examine the cytotoxicity of cilomilast, cell viability assay was performed in cultured NRK-49F cells using a CCK8 assay kit. With cilomilast treatment at increasing concentrations from 5 to 40 μM for 24 h, we found the concentration of cilomilast at 5 μM was safe for cells (Figure 5A). To define the effect of cilomilast on the profibrotic response in kidney cells, we added cilomilast to NRK-49F cells stimulated with TGF-β1. As shown by qRT-PCR and western blot, cilomilast treatment inhibited the expression of *Fn*, α*-SMA, Col-I*, and *Col-III* (Figures 5B–D). Immunofluorescence analysis demonstrated that the expression of FN was significantly reduced after treatment with cilomilast (Figure 5E). Similarly, the inflammatory response induced by TGF-β1 was also attenuated by cilomilast treatment (Figure 5F). These data revealed an antifibrotic role of cilomilast via the inhibition of renal fibroblast activation.

### Cilomilast Treatment Inhibited the Activation of TGF-β1-Smad2/3 Signaling

**Figure 6:**
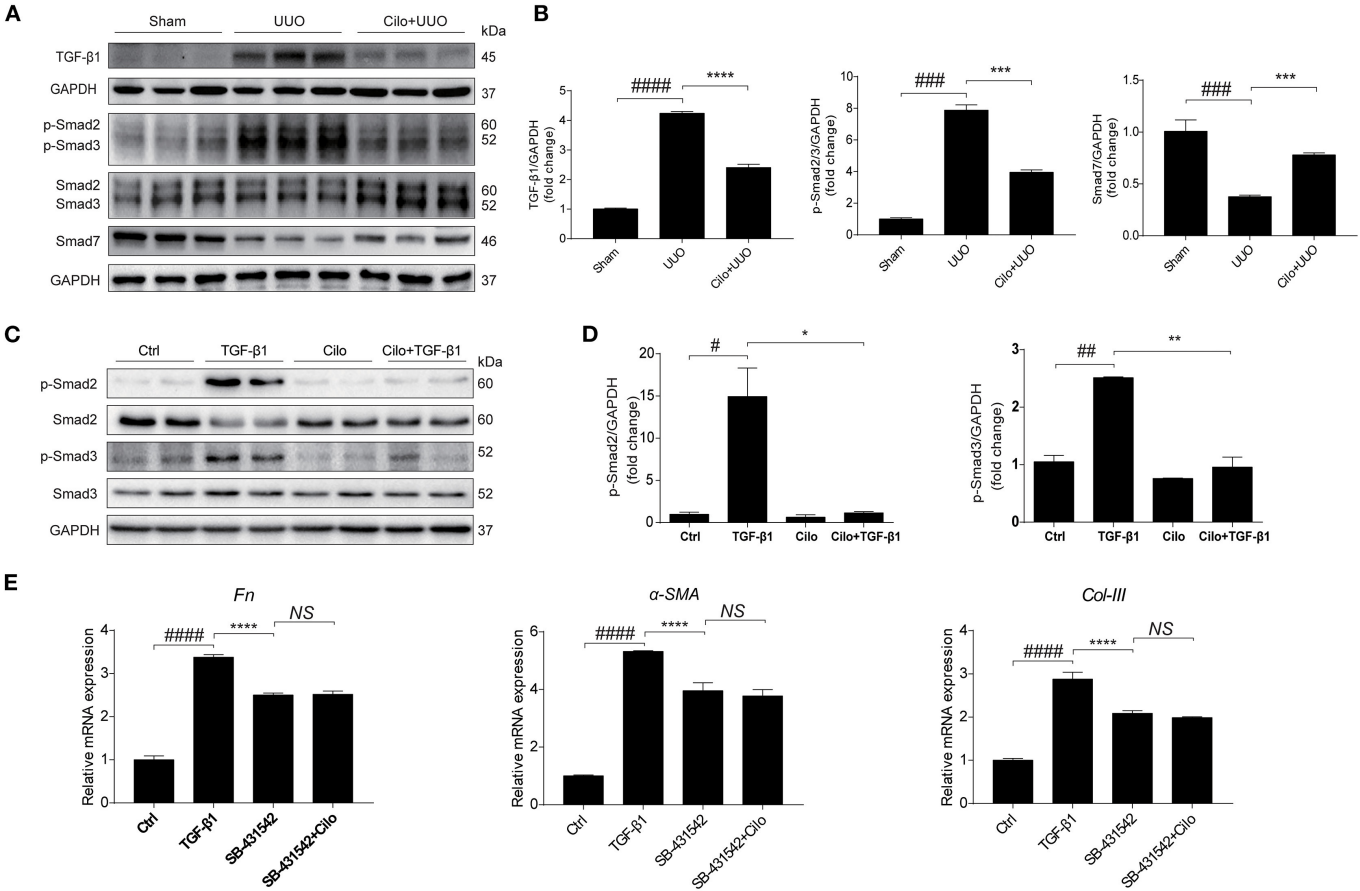
Cilomilast treatment inhibited the activation of TGF-β1-Smad2/3 signaling. **(A)** Western blot analyses of p-Smad2, Smad2, p-Smad3, Smad3, Smad7, and TGF-β1 protein expression in kidney tissues. **(B)** Densitometry of the western blot results in **(A)**. **(C)** Western blot analyses of p-Smad2, Smad2, p-Smad3, and Smad3 protein expression in NRK-49F cells. **(D)** Densitometry of the western blot results in **(C)**. **(E)** NRK-49F cells were pretreated with SB-431542 and cilomilast, and then incubated with TGF-β1 for 24 h. qRT-PCR analyses of *Fn*, α*-SMA*, and *Col-III* mRNA expressions in NRK-49F cells. The data are presented as the mean ± SEM. Statistically significant differences were determined by one-way ANOVA. ^#^*P* < 0.05, ^##^*P* < 0.01, ^###^*P* < 0.001, ^####^*P* < 0.0001, *P < 0.05, **P < 0.01, ***P < 0.001, ****P < 0.0001, NS, not significant.

TGF-β1-Smad2/3 signaling plays a critical role in renal interstitial fibrosis and inflammation (30). *In vivo*, we found cilomilast treatment inhibited the activation of Smad2/3 pathway in obstructed kidney tissues, as evidenced by decreased protein expression of TGF-β1, p-Smad2 and p-Smad3 and a significant increase in protein levels of Smad7 (Figures 6A,B). *In vitro*, western blot results showed that cilomilast could markedly decrease the expression of p-Smad2 and p-Smad3 in NRK-49F cells treated with TGF-β1 (Figures 6C,D). Furthermore, we used Smad2/3 inhibitor SB-431542 (1 μM) to explore whether cilomilast protected against TGF-β1-induced fibroblast activation through suppressing Smad2/3 pathway in this study. As shown in Figure 6E, when NRK-49F cells were pretreated with SB-431542 (1 μM), cilomilast failed to further ameliorate TGF-β1-induced fibroblast activation. Collectively, these results suggested that cilomilast may exert its antifibrotic effect by inhibiting the activation of TGF-β1-Smad2/3 signaling.

## Discussion

Renal tubulointerstitial fibrosis is the final outcome for all CKDs, leading to progression to end-stage renal failure (ESRD). To date, there are no effective therapeutic approaches in clinic, which results in a heavy socioeconomic burden. Thus, it is necessary to develop effective drugs for treating renal tubulointerstitial fibrosis. Based on the data from our study, cilomilast, a selective phosphodiesterase-4 inhibitor and phase III clinical drug, can remarkably reduce UUO-induced renal tubulointerstitial fibrosis and renal inflammation *in vivo* and *in vitro*. To the best of our knowledge, this is the first study to report that cilomilast may serve as a potent therapeutic agent for preventing the progression of renal tubulointerstitial fibrosis.

Factors that contribute to CKD progression include parenchymal cell loss, chronic inflammation, fibrosis and reduced regenerative capacity of the kidney (31). PDE4, which is a member of the PDE family, has four subtypes (PDE4A-PDE4D), and PDE4B plays an important role in inflammation (32). Cilomilast has been reported to treat cisplatin nephrotoxicity by antagonizing inflammation (12). Inflammation can lead to the progression of renal tubulointerstitial fibrosis (29). In the present study, the mRNA expression of proinflammatory factors was significantly reduced after cilomilast treatment. Immunohistology results revealed that cilomilast dramatically decreased the F4/80^+^ and TNF-α expression *in vivo*. Meanwhile, we confirmed that cilomilast downregulated the expression of fibrotic markers. Thus, cilomilast, as a selective phosphodiesterase-4 inhibitor, could block the inflammation to relieve renal tubulointerstitial fibrosis to some extent. KIM-1 and NGAL are considered to be important markers for evaluating renal tubular damage (33, 34). Recent studies indicated that the expression of KIM-1 and NGAL was significantly increased in UUO mice (35, 36). Our results revealed that cilomilast reversed renal tubular injury, which could contribute to improved tubular integrity and reduced subsequent pathology, such as inflammation and fibrosis.

Excessive deposition of ECM components in the renal interstitium could result in renal tubulointerstitial fibrosis (37). Smad2/3 signaling is closely associated with renal tubulointerstitial fibrosis (38), which can promote the fibrotic response by directly facilitating the production of ECM through its binding to specific promoter regions of collagen genes and the inhibition of ECM degradation (39, 40). TGF-β1 is a potent pathogenic factor of renal fibrosis, which could promote ECM production and renal tubulointerstitial fibrosis (41). The activation of TGF-β1 triggers the nuclear localization of Smad2/3 in tubular epithelial cells and fibroblasts (42). To exert its profibrotic role in kidney disease, TGF-β1 can act by stimulating Smad2/3 to positively or negatively regulate microRNAs (43, 44). Inhibiting Smad2/3 phosphorylation during TGF-β1-mediated epithelial-mesenchymal transition attenuated kidney fibrosis (45). In the contrast, up-regulating Smad2/3-related signaling pathway may enhance the progression of CKD (46). Indeed, Smad3-deficient mice were protected from UUO-induced renal tubulointerstitial fibrosis (47). In this study, we found that cilomilast could inhibit the expression of TGF-β1 induced by UUO and downregulated the phosphorylation levels of p-Smad2 and p-Smad3. The present study provided evidence that cilomilast has a protective effect on renal tubulointerstitial fibrosis possibly by downregulating the expression of TGF-β1, further inhibiting Smad2/3 phosphorylation.

In summary, we found that cilomilast remarkably attenuated renal tubulointerstitial fibrosis and inflammation in a CKD model of UUO. Cilomilast could decrease the expression of collagen, fibronectin and α-SMA, possibly by inhibiting TGF-β1-Smad2/3 pathway activation. Cilomilast is a selective phosphodiesterase-4 inhibitor that is currently in clinical trial for the treatment of COPD. Thus, the present study provided the rationale for further clinical trials to evaluate cilomilast in treating CKD.

## Data Availability Statement

The original contributions presented in the study are included in the article/supplementary material, further inquiries can be directed to the corresponding authors.

## Ethics Statement

The animal study was reviewed and approved by Nanjing Medical University Institutional Animal Care and Use Committee (registration number: IACUC-1809017).

## Author Contributions

XY and ZJ designed the experiment. MX, SL, XY, and JW performed the experiments and data analyses. XY, ZJ, and MX drafted the manuscript. ZJ, XY, YZ, SH, WG, and AZ revised and approved the manuscript. All authors contributed to the article and approved the submitted version.

## Conflict of Interest

The authors declare that the research was conducted in the absence of any commercial or financial relationships that could be construed as a potential conflict of interest.

## Abbreviations

CKD: chronic kidney disease
UUO: unilateral ureteric obstruction
ESRD: end-stage renal diseases
cAMP: cyclic adenosine monophosphate
COPD: chronic obstructive pulmonary disease
IHC: immunohistochemistry
ECM: extracellular matrix
PDE4: phosphodiesterase 4
TGF-β1: transforming growth factor-β1
FN: fibronectin
α-SMA: α-smooth muscle actin
Col-I: collagen I
KIM-1: kidney injury molecule-1
NGAL: neutrophil gelatinase-associated lipocalin
SEM: standard errors of the mean.
